# Supplementary material for: Predictors of Cardiometabolic Health a Few Months Postpartum in Women Who Had Developed Gestational Diabetes
Source: Nutrients. 2025 Jan 22;17(3):390. doi: 10.3390/nu17030390 (PMC11820877; doi:10.3390/nu17030390)
Supplement: Supplementary file 1 [file nutrients-17-00390-s001.zip › nutrients-3413591-supplementary.pdf]

Table S1. Baseline characteristics.

	All<br>n = 558	Normoglycaemia<br>n = 356 (63.8%)	Prediabetes<br>n = 174 (31.2%)	Type2 diabetes<br>n = 28 (5.0%)	p-value
**Demographic characteristics**					
Age (years)	35.6 (32-38.2)	35.4 (32-38.1)	35.8 (32.4-38.3)	34.7 (29.7-38.1)	0.433
Ethnicity					0.001
White	244 (43.7)	180 (50.6)	57 (32.8)	7 (25.0)	
Black	140 (25.1	73 (20.5)	59 (33.9)	8 (28.6)	
South Asian	99 (17.7)	61 (17.1)	29 (16.7)	9 (32.1)	
East Asian	48 (8.6)	29 (8.1)	17 (9.8)	2 (7.1)	
Mixed	27 (4.8)	13 (3.7)	12 (6.9)	2 (7.1)	
First or second degree family history of diabetes	276 (49.5)	169 (47.5)	94 (54.0)	13 (46.4)	0.478
Parity					0.002
Nulliparous	234 (41.9)	159 (44.7)	57 (32.8)	18 (64.3)	
Parous	324 (58.1)	197 (55.3)	117 (67.2)	10 (35.7)	
Parous with previous GDM	92 (28.4)	47 (23.9)	45 (38.5)	6 (60)	<0.001
Method of conception					0.718
Spontaneous	495 (88.7)	311 (87.4)	159 (91.4)	25 (89.3)	
Ovulation induction	4 (0.7)	3 (0.8)	1 (0.6)	0 (0)	
In vitro fertilization	59 (10.6)	42 (11.8)	14 (8.0)	3 (10.7)	
BMI at 11-13 weeks' gestation (kg/m^2)	27.9 (23.8-33.0)	27.6 (23.5-32.5)	28.7 (23.9-34.2)	26.8 (31.9-34.9)	0.092
BMI at 36 weeks (kg/m^2)	31.0 (27.2-35.2)	30.9 (27.0-34.6)	31.5 (27.3-36.3)	32.9 (28.8-39.4)	0.424
**Pregnancy outcome**					
Gestational age at delivery (weeks)	39 (38.3-39.6)	39.1 (38.4-39.7)	38.9 (38.0-39.3)	39.1 (38.2-39.8)	0.049
Birthweight percentile	49.6 (21.1-74.8)	56.1 (22.5-76.4)	39.1 (20.6-70.1)	47.8 (19.7-78.1)	0.001
Treatment for GDM					<0.001
Diet	209 (37.5)	161 (45.2)	44 (25.3)	4 (14.3)	
Metformin	228 (40.9)	138 (38.8)	78 (44.8)	12 (42.9)	
Insulin (+/- metformin)	121 (21.7)	57 (16)	52 (29.9)	12 (42.9)	
Gestational age at diagnosis of GDM					<0.001
< 24 weeks	113 (20.7)	41 (11.7)	55 (33.7)	15 (53.6)	
≥ 24 weeks	433 (79.3)	308 (88.3)	112 (66.3)	13 (46.4)	
**Postnatal visit**					
Postnatal BMI (kg/m^2)	28.2 (24.0-32.8)	27.6 (23.6-31.6)	29.5 (24.8-34.3)	29.0 (24.6-36.2)	0.025
Antihypertensive medication	7 (1.3)	2 (0.6)	4 (2.3)	1 (3.6)	0.127
Medications associated with an increased risk of hyperglycaemia*	19 (3.4)	12 (3.4)	7 (4.0)	0 (0)	0.551

Quantitative variables expressed median and IQR (p25-p75). Qualitative variables expressed as number of cases (%).

*Medications associated with increased risk of hyperglycaemia were systemic glucocorticoids, selective serotonin reuptake inhibitors, antipsychotics and antiretroviral.

Table S2. Predictors of dysglycaemia according to ADA criteria.

Predictor	Univariable	Multivariable

	OR (95% CI)		OR (95% CI)	
**Demographic characteristics**				
Age - ≥36 years	1.05 (0.75-1.46)	0.785		
Ethnicity				
White (reference)	1.00		1.00	
Black	4.37 (2.77-6.88)	<0.001	3.71 (2.26-6.10)	<0.001
South Asian	1.81 (1.13-2.90)	0.014	1.67 (1.00-2.78)	0.050
East Asian	2.39 (1.27-4.51)	0.007	3.02 (1.47-6.19)	0.003
Mixed	1.96 (0.88-4.37)	0.100	2.12 (0.91-4.93)	0.081
First or second degree family history of diabetes	1.19 (0.85-1.66)	0.302		
Parity				
Parous (reference: nulliparous)	0.87 (0.60-1.26)	0.460		
Previous GDM (reference: parous without previous GDM)	1.49 (0.92-2.41)	0.106	1.68 (0.94-3.00)	0.078
Method of conception				
Spontaneous (reference)	1.00			
Ovulation induction	0.28 (0.03-2.71)	0.272		
In vitro fertilization	0.62 (0.36-1.07)	0.084		
BMI at 11-13 weeks' gestation - ≥28 kg/m^2	1.84 (1.32-2.58)	<0.001		
BMI at 36 weeks - ≥31 kg/m^2	1.47 (1.05-2.05)	0.024		
**Pregnancy outcome**				
Gestational age at delivery - ≥39 weeks	1.14 (0.82-1.60)	0.440		
Birthweight percentile - >50 percentile	1.49 (1.06-2.08)	0.020	1.57 (1.06-2.32)	0.025
Treatment for GDM				
Diet (reference)	1.00			
Metformin	1.81 (1.25-2.62)	0.002		
Insulin (+/- metformin)	2.80 (1.65-4.75)	<0.001		
Gestational age at diagnosis of GDM - < 24 weeks	2.84 (1.80-4.47)	<0.001	2.09 (1.23-3.56)	0.006

**Postnatal visit**		
Postnatal BMI - ≥28 kg/m^2	2.10 (1.50-2.95)	<0.001
Breastfeeding (reference: yes)	1.07 (0.76-1.50)	0.703
Antihypertensive medication (reference: no)	2.25 (0.43-11.70)	0.335
Medications associated with an increased risk of diabetes (reference: no)	0.99 (0.40-2.48)	0.983
Waist circumference - ≥91 cm	1.95 (1.39-2.74)	<0.001
Serum triglycerides - ≥0.9 mmol/L	1.34 (0.96-1.88)	0.085
Serum HDL-Cholesterol - <1.5 mmol/L	1.35 (0.96-1.89)	0.083
Systolic blood pressure - ≥117 mmHg	1.42 (1.02-1.99)	0.039
Diastolic blood pressure - ≥74 mmHg	1.24 (0.89-1.72)	0.214

Continuous variables were dichotomized as lower than median and greater than or equal to the median. Multivariable model contains only those variables which had p<0.1 in the backward elimination. The p-values from backward elimination are not shown.
